# C-reactive protein/oxidised low-density lipoprotein/β2-glycoprotein I complex promotes atherosclerosis in diabetic BALB/c mice via p38mitogen-activated protein kinase signal pathway

**DOI:** 10.1186/1476-511X-12-42

**Published:** 2013-03-26

**Authors:** Rui Zhang, Sai-Jun Zhou, Chun-Jun Li, Xiao-Nan Wang, Yun-Zhao Tang, Rui Chen, Lin Lv, Qian Zhao, Qiu-Ling Xing, De-Min Yu, Pei Yu

**Affiliations:** 1 Department of Diabetic Nephropathy Hemodialysis, Key Laboratory of Hormones and Development (Ministry of Health), Metabolic Diseases Hospital & Tianjin Institute of Endocrinology Tianjin Medical University, Tongan Street, Tianjin, Heping District, 300070, China; 2 Department of Hand Microsurgery, Tianjin Hospital, Tianjin, 300070, China

**Keywords:** Diabetes, Atherosclerosis, CRP/oxLDL/β2GPI complex, p38MAPK, BALB/c mice

## Abstract

**Background:**

The aim of this study was to investigate the effect of C-reactive protein/oxidised low-density lipoprotein/β2-glycoprotein I (CRP/oxLDL/β2GPI) complex on atherosclerosis (AS) in diabetic BALB/c mice.

**Methods:**

BALB/c mice were fed high-fat and normal diet. Eight weeks later, the mice fed with high-fat diet were injected with streptozotocin (STZ) to induce diabetes. The diabetic mice were respectively injected twice monthly with 20 μg oxLDL, 20 μg β2GPI, 40 μg oxLDL/β2GPI complex, 44 μg CRP/oxLDL/β2GPI complex, and PBS. Aortas were stained with Sudan IV to investigate lipid plaque formation. The infiltration condition of smooth muscle cells (SMCs), macrophages, and T cells in the aortas were determined by immunohistochemistry (IH). The mRNA expressions of receptors associated with lipid metabolism were quantified by real-time PCR. The phosphorylation of p38 mitogen-activated protein kinase (p38MAPK) and MKK3/6 in aorta tissues were assessed by Western blot. The expression of inflammation cytokines was evaluated by protein chip.

**Results:**

The lipid plaques were more extensive, the lumen area was obviously narrower, the ratio of intima and media thickness were increased, and the normal internal elastic lamia structure and endothelial cell disappeared (*P* < 0.05) in the oxLDL and CRP/oxLDL/β2GPI groups (*P* < 0.05). CRP/oxLDL/β2GPI complex dramatically promoted infiltration of SMCs, macrophages, and T cells, improved the mRNA expression of ABCA1 and ABCG1, but reduced the mRNA expression of SR-BI and CD36 and increased the phosphorylation of p38MAPK and MKK3/6 (all *P* < 0.05). The highest expression levels of IL-1, IL-9, PF-4, bFGF, and IGF-II were detected in the CRP/oxLDL/β2GPI group (*P* < 0.05).

**Conclusions:**

CRP/oxLDL/β2GPI complex aggravated AS in diabetic BALB/c mice by increasing lipid uptake, the mechanism of which may be mediated by the p38MAPK signal pathway.

## Background

Atherosclerosis (AS) is the main etiology of diabetic macroangiopathy and the leading cause of disability and death of patients with diabetes mellitus [1]. Diabetic patients with cardiovascular diseases (CVD) possess more diffused AS lesions with more severe degrees compared with other CVD patients [2,3], suggesting that diabetic AS has a unique pathogenic mechanism. β2-glycoproteinI (β2GPI), a highly glycosylated plasma protein with an approximate molecular weight of 50 kDa, was found to be the main antigen in the serum of anti-phospholipid syndrome (APS) patients [4]. Β2GPI interacts with oxidised low density lipoprotein (oxLDL) via 7-ketocholesterol having an w-carboxyl acyl chain, producing stable and nondissociable oxLDL/β2GPI complexes [5], which could further interacts with C-reactive protein (CRP), producing CRP/oxLDL/β2GPI complex [6]. Recent studies have demonstrated that β2GPI complexes (oxLDL/β2GPI and CRP/oxLDL/β2GPI) have a close relationship with AS [6,7]. Research has demonstrated that the levels of oxLDL/β2GPI and CRP/oxLDL/β2GPI complexes in the serum of diabetic patients were obviously higher than those of the normal control [6]. The amounts of these complexes have been found in AS plaque tissues using immunohistochemistry, indicating that β2GPI complexes have an important function in AS development [6,7]. In addition, Matsuura et al. discovered that the CRP/oxLDL/β2GPI complex was significantly elevated only in diabetic AS patients [7], indicating that this complex is specific to diabetic AS but the pathogenic mechanism is not clear. Thus, this study further explored the effect of CRP/oxLDL/β2GPI complex on AS occurrence and on the development of diabetic mice through in vivo research and the possible pathogenic mechanism.

## Results

### Blood glucose and body weight

**Figure 1:**
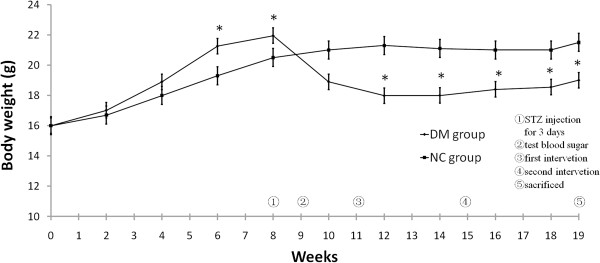
**Body weight of DM group and NC group.** ●, DM group (n=120); ■, NC group (n=24). *P < 0.05, compared with group NC. Maintained on high-fat and sugar diet and standard chow diet, respectively, mice in DM group and NC group were weighed every week. Eight weeks later, DM group was intraperitoneally injected with 80 mg/kg 2% STZ three times for three consecutive days. The tail vein blood glucose was measured 72 h after the injection, and those with blood glucose ≥16.7 mmol/L were considered DM mice. The NC group was simultaneously injected with sodium citrate buffer. Two weeks later, the DM group was treated with oxLDL 20 μg, β2GPI 20 μg, oxLDL/β2GPI complex 40 μg, CRP/oxLDL/β2GPI complex 44 μg, and PBS, after which they were randomly divided into oxLDL group, β2GPI group, oxLDL/β2GPI group, CRP/oxLDL/β2GPI group, and PBS group, respectively (each group n=24). The same interventions were boosted four weeks later. NC group were injected with PBS at the same time. Four weeks later, blood was obtained via retro-orbital plexus and then sacrificed by cervical dislocation.

After STZ injection, the mice in the DM group all became diabetic, but no animal died until they were sacrificed. After the mice were fed with high-fat and sugar diet for eight weeks, the body weight of the DM group became noticeably higher, which was different from that of group NC (*P*<0.05). The DM group significantly lost weight after STZ injection for three weeks compared with the NC group (*P*<0.05), then gradually gained weight because of continuous subsistence on high-fat diet (Figure 1). The blood glucose of the DM group was three times higher than that of the NC group (*P*<0.01).

### Plasma lipid level of diabetic BALB/c mice

**Figure 2:**
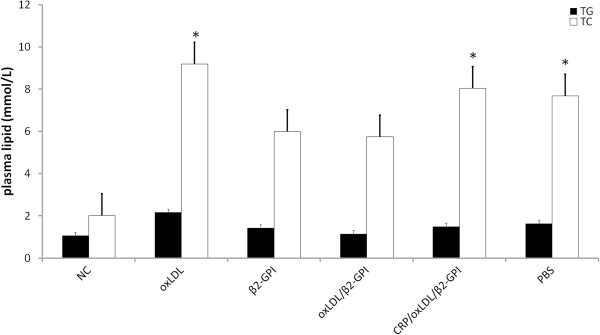
**Plasma lipid level of the DM group significantly increased.** *P < 0.05, compared to group NC.

Plasma triglycerol (TG) and total cholesterol (TC) levels were higher in DM group than in NC group. The TC levels of oxLDL, CRP/oxLDL/β2GPI, and PBS groups were higher than those of NC group (*P*< 0.05), whereas the TG level did not exhibit significant difference in all the DM groups (Figure 2). The β2GPI and oxLDL/β2GPI groups showed decreased levels of plasma TG and TC.

### CRP/oxLDL/β2GPI promotes the formation of atherosclerotic plaque in aortas

**Figure 3:**
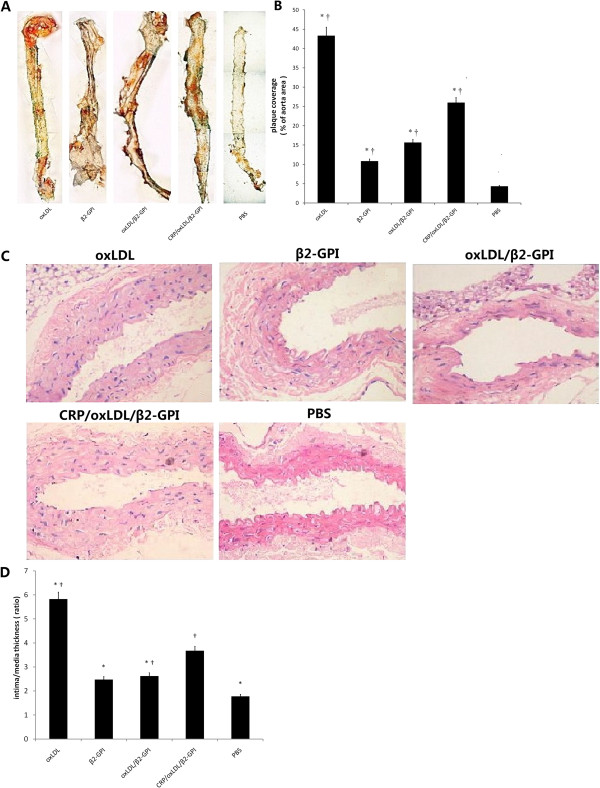
**Histological quantitation of the AS lesion in aortas.** After induction of diabetes and treatment with 20 μg oxLDL, 20 μg β2GPI, 40 μg oxLDL/β2GPI complex, 44 μg CRP/oxLDL/β2GPI complex and PBS (as described in the Methods section), aortas were removed, longitudinally cut open,stained with Sudan IV, or paraffin-embedded, sectioned, deparaffinated and then stained with haematoxylin and eosin. The quantitation of the AS lesion and intima thickness were measured using Image Pro-Plus 6.0. The percentage of the plaque area to the total aorta area and the ratios of intima/media thickness of lesions at the aortic roots were calculated. Results are shown as means ± SEM. **A**: Representative images of Sudan IV stain(4×). **B**: Percentage of plaque area to total aorta area (n=4 mice per group). *P < 0.05, compared with the CRP/oxLDL/β2GPI group, ^†^*P* < 0.05, compared with the PBS group. **C**: Representative H&E stain at the root of the aortas, 200× magnification. **D**: The ratios of intima/media thickness of lesions at the aortic roots (n=4). *P < 0.05, compared with the CRP/oxLDL/β2GPI group, ^†^*P* < 0.05, compared with the PBS group.

Pale yellow wax-like hills were spread along the aortic intima of the DM group, which were lined with lipid streak and atherosclerotic plaque. The entire aortic intima was uneven with a stiff wall. Tissues of the DM group seemed crisper and more difficult to extend, except those in the PBS group. Among all the DM groups, PBS group possessed the least lipid deposits, whereas CRP/oxLDL/β2GPI group exhibited relatively obvious AS plaques, ranking second to the oxLDL group (Figure 3A). As seen in Figure 3B, the lesion in the CRP/oxLDL/β2GPI group was about 2.4-fold that of the β2GPI group (*P*<0.05), 1.66-fold that of the oxLDL/β2GPI group (*P*<0.05), and 6-fold that of the PBS group (*P*<0.05).

### CRP/oxLDL/β2GPI increases intima/media thickness value in aortas

H&E stained cross-sections at the root of the aortas showed different extents of damage in the DM group. Only the internal elastic lamina of the PBS group remained almost intact with minimal intima proliferation. However, the structure of the intima in the other DM groups seemed disordered, with intima proliferation and media atrophy. Moreover, almost no normal structure was observed in the CRP/oxLDL/β2GPI group. Endothelial cells fell off and the normal structure of the internal elastic lamina disappeared (Figure 3C). As seen in Figure 3D, ratios of intima/media thickness (IMT, thickness of intima and media were measured at the thickest part of the plaques) of the oxLDL group and the CRP/oxLDL/β2GPI group remarkably increased (*P* < 0.05).

### CRP/oxLDL/β2GPI promotes the infiltration of SMCs, macrophages and T cells in the intima of aortas

**Figure 4:**
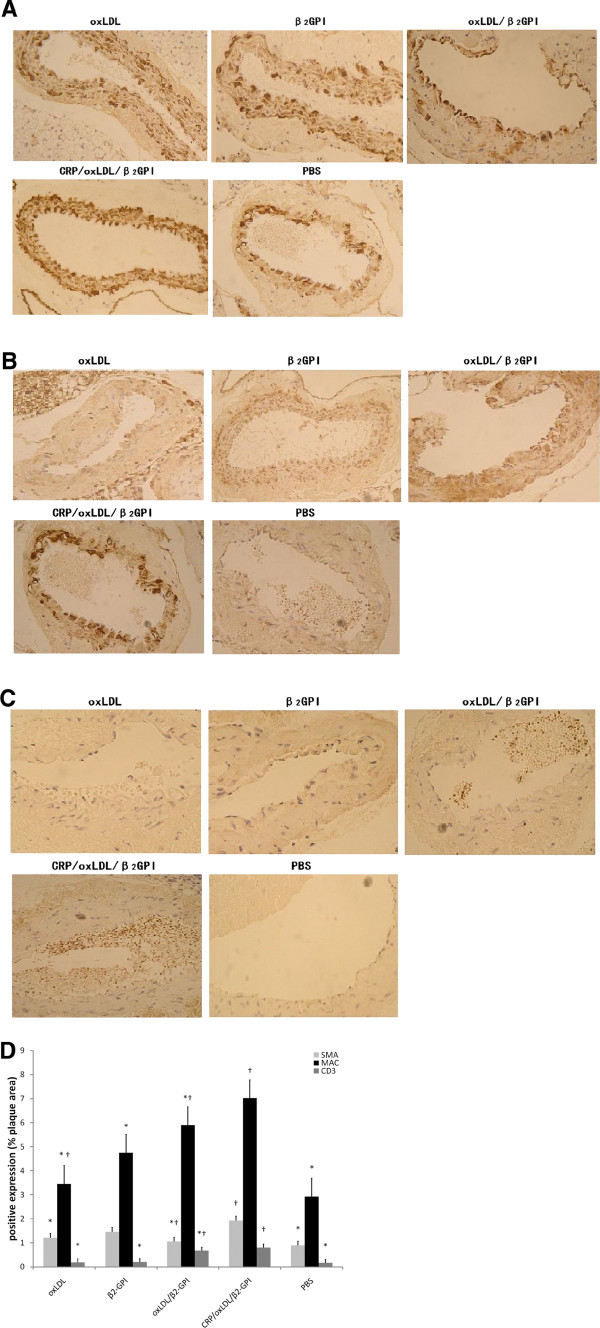
**Effect of CRP**/**oxLDL**/**β2**-**GPI on infiltration of inflammatory cells in the aortas.** Aortas were removed, paraffin-embedded, deparaffinated, underwent antigen retrieval, blocked, and then incubated with primary antibodies (Mac 1:200 or α- SMA 1:100 or CD3 1:100) at 4°C overnight. Biotinylated secondary antibody (1:200) was added, followed by HRP-labeled streptavidin incubation. Infiltrations of macrophages, SMCs and T cells in the aortas were quantified as the percentage of the luminal surface covered by positive cells to the plaque area using Image Pro-Plus 6.0. Results are shown as means ± SEM. **A**: Infiltration of SMCs in the aortas, 400× magnification. **B**: Infiltration of macrophages in the aortas, 400× magnification. **C**: Infiltration of T cells in aortas, 400× magnification. **D**: Positive expressions of SMA, MAC and CD3 (n=4), expressed as the percentage to plaque area. *P <0.05, compared with the CRP/oxLDL/β2GPI group, †*P* < 0.05, compared with the PBS group.

CRP/oxLDL/β2GPI group showed strong positive expression of α-SMA in each layer of aorta. The other DM groups only displayed positive expression on the surface of the intima, not on the shrink media or not as strong as that in the CRP/oxLDL/β2GPI group (Figure 4A). CRP/oxLDL/β2GPI group showed remarkable positive areas of macrophages (Figure 4B). T cell expression was not as obvious as those of SMC and macrophages, but certain amounts of T cells were found in the lumen in the CRP/oxLDL/β2GPI group. Moreover, positive CD3 expression was not limited to the intima, but was also found on the adventitia (Figure 4C). Using Image Pro-Plus 6.0, the α-SMA, MAC, and CD3 positive expressions of AS lesion in the CRP/oxLDL/β2GPI group were found to be 1.925, 7.025, and 0.8025, respectively, which were higher than those in other DM groups (*P*<0.05). By contrast, the expressions of the three kinds of cells in the PBS group were much less than those of the others (Figure 4D).

### CRP/oxLDL/β2GPI increases ABCA1 and ABCG1expressions and decreases CD36 and SRBI expressions in aortas

**Figure 5:**
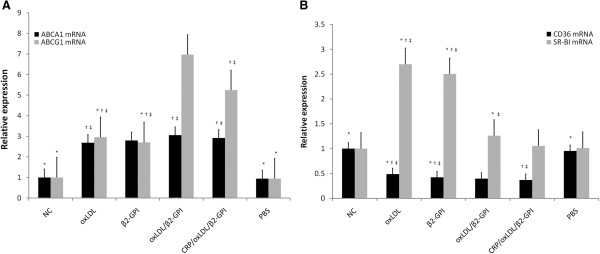
**Effect of CRP**/**oxLDL**/**β2GPI on receptors associated with lipid metabolism gene expression in the aortas.** After induction of diabetes and treatment with oxLDL, β2GPI, oxLDL/β2GPI complex, CRP/oxLDL/β2GPI complex and PBS (as described in the Methods section), aortas were removed and the expression of selected genes was measured using real-time PCR. Estimates of the quantity of the PCR product were obtained by densitometry using the Quantity One analysis software package. Results are show as means ± SEM of mRNA level normalized to β-actin in the normal (non-diabetes) group. **A**: ABCA1 mRNA and ABCG1 mRNA expression level in the aortas. **B**: CD36 mRNA and SR-BI mRNA expression level in the aortas. Expression level of NC group was normalized as 1, n= 4. *P < 0.05, compared with the CRP/oxLDL/β2GPI group, †*P* < 0.05, compared with the PBS group, ‡*P*< 0.05, compared with the NC group.

Real-time PCR results showed that the PBS and NC groups seemed to share the same relative expression level of all the target mRNAs. The other DM groups presented the opposite tendency, with significantly up-regulated mRNA level of ATP-binding cassette transporter protein A1 (ABCA1) and ATP-binding cassette transporter protein G1 (ABCG1) (Figure 5A) and down-regulated CD36 mRNA expression level (Figure 5B). The CRP/oxLDL/β2GPI group obviously promoted ABCG-1 mRNA expression and suppressed CD36 mRNA expression compared with the oxLDL group (*P*<0.05). However, scavenger receptor type B1 (SR-BI) mRNA expression of the CRP/oxLDL/β2GPI group was almost the same as that of the PBS and NC groups, with remarkably depressed SR-BI compared with the other DM groups (*P* < 0.05) (Figure 5B).

### CRP/oxLDL/β2-GPI increases phosphorylation of p38MAPK and MKK 3/6 expressions in aortas

**Figure 6:**
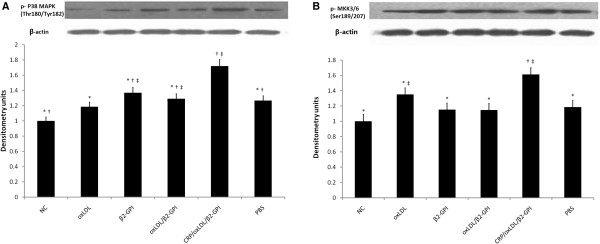
**Effects of CRP**/**oxLDL**/**β2GPI on P38MAPK**-**associated signaling in the aortas.** Extracts of aortas from NC and diabetes animals were subjected to Western blots using the anti-bodies shown. **A**: Phosphorylation level of p38MAPK in aortas, expressed as the ratio to NC group (expression level of NC group was normalized as 1, n= 4). *P < 0.05, compared with the CRP/oxLDL/β2GPI group, ^†^*P* < 0.05, compared with the PBS group, ‡*P*< 0.05, compared with the NC group. **B**: Phosphorylation level of MKK3/6 in aortas, expressed as the ratio to NC group (expression level of NC group was normalized as 1, n= 4). *P < 0.05, compared with the CRP/oxLDL/β2GPI group, †*P* < 0.05, compared with the PBS group, ^‡^*P*< 0.05, compared with the NC group.

As shown in Figure 6, the CRP/oxLDL/β2GPI group significantly triggered the phosphorylation level of p38MAPK and MKK 3/6 (*P* < 0.05) to a greater extent compared with the other groups. Unlike CRP/oxLDL/β2GPI, β2GPI and oxLDL/β2GPI evoke similar effects on p38MAPK phosphorylation with PBS.

### CRP/oxLDL/β2GPI increases bFGF, IGF-α, IL-1α, IL-9 and PF-4 activation in aortas

**Figure 7:**
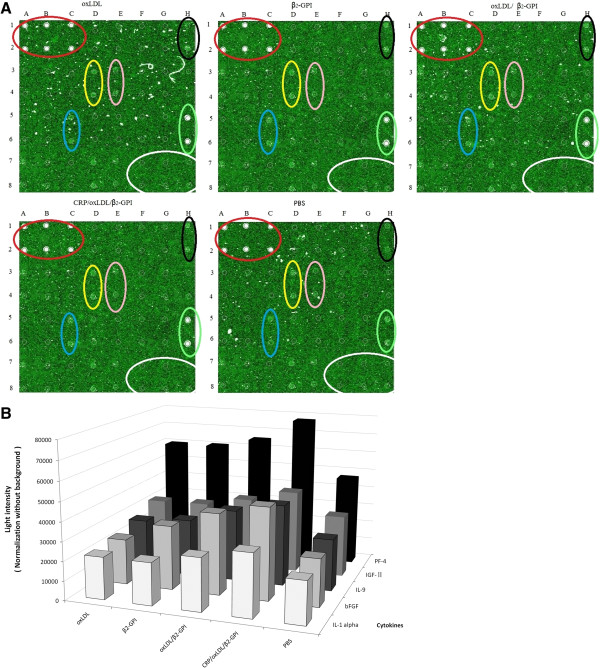
**Effects of CRP**/**oxLDL**/**β2GPI on inflammatory cytokines in the aortas.** Extracts of protein from the NC and diabetes animals were added into each well of Protein chip. A laser scanner (Scan-array Gx, PerkinElmer) was used to read the signals for each spot. The relative fold differences in the cytokine amount were determined by an analysis tool (Raybio, USA). **A**: Protein chip images. Red: positive control; black: bFGF; yellow: IGF-II; pink: IL-1; blue: IL-9; green: PF-4; white: negative control. **B**: IL-1, IL-9, bFGF, IGF-II and PF-4 average relative optical density in the aortas.

The mice protein antibody chip detected 24 kinds of cytokines. Each cytokine was represented by duplicate spots, as seen in Table 1. According to the chemiluminescence results, five cytokines were detected (Figure 7A) as follows: basic fibroblast growth factor (bFGF), insulin-like growth factor α (IGF-α), IL-1α, IL-9, and platelet factor-4 (PF-4). Among them, PF-4 was the most predominant. Values were obtained using LabWorks software. These five cytokines were induced to higher levels in the oxLDL/β2GPI group, ranking second to the CRP/oxLDL/β2GPI group (Table 2 and Figure 7B). Average light intensity was normalised without background for each pair of cytokine spots detected using Image Pro-Plus software. Results were represented as bars (n = 3 mice per group).

**Table 1:** Cytokines locations on the protein chip

	**A**	**B**	**C**	**D**	**E**	**F**	**G**	**H**
1	POS1	POS2	POS3	NEG	NEG	Eotaxin	Fasligand	bFGF
2	POS1	POS2	POS3	NEG	NEG	Eotaxin	Fasligand	bFGF
3	G-CSF	GM-CSF	IFN-γ	IGF-II	IL-1α	IL-1β	IL-12p40/p70	IL-12p70
4	G -CSF	GM-CSF	IFN-γ	IGF-II	IL-1α	IL-1β	IL-12p40/p70	IL-12p70
5	IL-13	IL-6	IL-9	Leptin	MCP-1	M-CSF	MIG	PF-4
6	IL-13	IL-6	IL-9	Leptin	MCP-1	M-CSF	MIG	PF-4
7	TIMP-1	TIMP-2	TNFα	Thrombopoietin	VEGF	NEG	NEG	NEG
8	TIMP-1	TIMP-2	TNFα	Thrombopoietin	VEGF	NEG	NEG	NEG

POS: positive control, NEG: negative control.

**Table 2:** IL-1, IL-9, bFGF, IGF-II and PF-4 average relative optical density in aortas

	**oxLDL**	**β2GPI**	**oxLDL/β2GPI**	**CRP/oxLDL/β2GPI**	**PBS**
IL-1α	21904	21907	27224	32273	21904
bFGF	23628	33264	42119	47649	24564
IL-9	27224	29689	37154	42225	26869
IGF-II	32189	32689	37154	43219	32189
PF-4	60035	60535	65535	77560	47084

## Discussion

CVD is a leading cause of the mortality in diabetic patients, and its remarkable pathological change is AS. Given the recent study of β2GPI complexes, the present study explored the role of β2GPI complexes on AS in the STZ-induced diabetic AS model.

This study successfully developed an animal model that acquired the properties of diabetic AS, that was higher blood sugar level, lipid level and intuitive lesion staining with Sudan IV, after atherogenic diet for 19 weeks and STZ injection.

Increased intima-media thickness (IMT) reflects cardiovascular risk, and its increasing value (at or above 1 mm) represents increased risk of myocardial infarction and/or cerebrovascular disease [8]. Our results, demonstrated that CRP/oxLDL/β2GPI treatment aggravated lipid deposition, increased IMT value in STZ-induced diabetic AS, promoted AS development and the risk of CVD.

Macrophages, SMCs and T cells have been considered to be the key cells involved in AS lesion development. Macrophages evolve into foam cells after amounts of lipid uptake and finally leading to early fatty streak formation. T cells, the main cells in cellular immunity, reach intima at an early stage of AS formation. Activated T cells produce cytokines, taking part in regulating lesion formation [9]. Meanwhile, As atherosclerosis progresses, vascular SMCs are in proximity to and physically interact with inflammatory cell types, e.g. monocytes and macrophages, which play a very important role in further exacerbating the disease [10]. Furthermore, histological images have shown that prior to the onset of complex plaque development in human tissues [11,12] and experimental models of atherosclerosis, markers of inflammation are observed in SMCs in the media of the vessel wall, such as VCAM-1 and inflammatory transcriptional mediators, such as activated NF-κB [13,14].

Immunohistochemistry results confirmed stronger positive expressions and more infiltration of SMCs, macrophages and T cells along the intima of aortas in DM groups, especially after being treated with CRP/oxLDL/β2GPI.

Although much of the researches on atherosclerosis has focused on the intimal accumulation of lipids and inflammatory cells, there is an increasing amount of interest in the role of the adventitia in coordinating the immune response in atherosclerosis [15]. From the T cells and macrophage stain results (Figure 4), we found parts of positive expressions in the intima and in the adventitia in CRP/oxLDL/β2GPI group, but not in the other groups. Research has found that adventitia inflammatory cell infiltration occurs earlier than AS lesion formation [15], which indicating CRP/oxLDL/β2GPI could cause an early changes detected in diabetic AS.

ABCA1 and ABCG1 belong to cholesterol transporters in human pancreatic β cells, that play an important role in protecting against AS by facilitating cholesterol extracellular efflux from macrophages or foam cells [16]. Moreover, recent studies have found that the effect of ABCG1 on AS lesion development in LDLR^-/-^ mice depends on the stage of AS, that is, ABCG1 is atheroprotective in early lesions. As the lesions advance, ABCG1 enhances apoptosis and the compensatory mechanism, thus promoting further AS progression [17]. In addition, ABCA1 high expression was associated with plaque complexity and oxLDL high level in plaques. ABCA1 mRNA and protein expressions exhibited an increasing trend before AS formation. oxLDL induced ABCA1 expression, and the foam cells induced by oxLDL could also enhance ABCA1 expression [18]. Our study found that mRNA expressions of ABCA1 and ABCG1 in aortas of diabetic Balb/c mice were elevated in the CRP/oxLDL/β2GPI group. Therefore, the capability of CRP/oxLDL/β2GPI complex to increase ABCA1 and ABCG1 mRNA expression may be one of the important mechanisms of pro-AS.

Moreover, not only cholesterol metabolism is involved in the pathological change of AS, lipoprotein receptors, especially scavenger receptors, participate in AS process directly.

Scavenger receptor-B includes two subtypes, CD36 and SR-BI, which ligands are oxLDL and HDL, respectively. Combined with CD36, oxLDL, subsequently stimulates macrophage infiltration and SMC migration and proliferation, leading to vascular endothelial cells apoptosis, erosion of vessel walls, destroyed coagulation mechanism, and finally macrophage apoptosis, resulting in AS plaques [19]. CD36 inhibition of CRP/oxLDL/β2GPI may be a result of compensatory mechanism at the early stage of AS lesion. On the other hand, SR-BI is the only membrane receptor that mediates the interaction between cells and HDL, preventing free cholesterol from accumulating at the artery walls, thus reducing AS incidence [20]. Lowering SR-BI expression through the CRP/oxLDL/β2GPI complex consequently leads to cholesterol accumulation in the macrophages and foam cell formation, both of which may take part in the pro-AS process.

It is known that β2GPI interacts with oxLDL via 7-ketocholesterol having an w-carboxyl acyl chain, producing stable and nondissociable oxLDL/β2GPI complexes [5,21,22].

Once β2GPI binds with oxLDL, specific epitope of oxLDL was covered, distrubing its binding with antibodies, thus also ameliorating the pro-atherosclerosis of oxLDL [23]. Furthermore, β2GPI decreased cellular accumulation of cholesterol via a reduction in cholesterol influx and an increase in cholesterol efflux, suggesting that β2GPI might play an important role in the prevention of atherosclerosis [24], and that may explain β2GPI and oxLDL/β2GPI made less lipid accumulation than oxLDL. However, when oxLDL/β2GPI further interacts with CRP through another site named oxPC [25], the pro-AS function of CRP/oxLDL/β2GPI complex was enhanced as our results showed, maybe due to the strong pro-inflammation function of CRP in AS of both cardiovascular and cerebrovascular diseases [26].

When refering to the signaling pathway potentially involved in AS pathogenesis, hyperglycemia AS causes activation of MAPK in the aortas of BALB/c mice. The major members of MAPKs found in aortas include p38 MAPK and are most strongly activated by oxidative stress, hyperglycemia and proinflammatory cytokines [27].

Our results demonstrated enhanced activation p38MAPK and its upstream protein p-MKK3/6 with the treatment of CRP/oxLDL/β2GPI complex in diabetic Balb/c mice. Hence, the p38MAPK pathway activated by the CRP/oxLDL/β2GPI complex may be the one of the key molecule mechanisms of pro-AS.

The protein chip was also applied in the investigation to quickly detect cytokines associated with AS development. We found that the expression level of five cytokines, namely, bFGF, IGF-II, IL-1α, IL-9, and PF-4 were significantly elevated. The five specific cytokines were expressed highest in the CRP/oxLDL/β2GPI group. All of them are participating in the regulation of AS onset and drive AS development through different ways [28-31], which prove that the CRP/oxLDL/β2GPI complex increases the inflammation level in diabetic Balb/c mice by increasing pro-inflammatory cytokine expression, leading to further AS development. However, the level of cytokines with elevated expression needs further verification by Western blot or enzyme-linked immunosorbent assay.

This study focused on the CRP/oxLDL/β2GPI complex, and explored and compared its effects with those of oxLDL, β2GPI, and oxLDL/β2GPI complexes on the genesis and progression of diabetic atherosclerosis. After the invention of the CRP/oxLDL/β2GPI complex, more AS plaques and lesions were elicited in diabetic Balb/c mice, which indicated the importance of CRP/oxLDL/β2GPI in the development of diabetic atherosclerosis and provided new evidence that p38MAPK is a principal regulator of inflammatory response.

## Materials and methods

### Reagents

oxLDL was purchased from XINYUANJIAHE Biotechnology Co., Ltd, BeiJing, China. CRP (pro-557) was from Protein Specialists, Israel. β2GPI was extracted and purified by TianJin Medical University Metabolic Diseases Hospital and Novo Nordisk (Details refer to Supplementary). STZ (s0130) were obtained from Sigma-Aldrich, USA. Anti-β2GPI MAb (WB-CAL-1), anti- apoB-100 MAb (N2E10) and anti-CRP Abs were gifts from professor Steven Krilis the Department of Medicine,St. George Hospital Clinical School, University of New South Wales, Sydney, Australia.

Rats anti-macrophage antibody (MAC, sc-101447), mice anti-β-actin antibody, mice anti-phospho-MKK3/MKK6 (9231s) antibody were from Santa Cruz Biotechnology, Inc, USA. Mice anti-α-smooth muscle actin (SMA, BM0002) antibody, rabbit anti-CD3 (BA0429) antibody were purchased from Boster, Wuhan, China. Rabbit anti-phospho-p38MAPK (9212) antibody was purchased from Cell Signaling Technology, USA. Cytokine array kit (Mouse angiogenesis antibody array G Series 1) was gained from RayBiotech Inc, USA.

### Animals

Eight-week old female Balb/c mice bought from Peking University Laboratory Animal Center (BeiJing, China) were housed in a temperature-controlled animal facility under a 12 h light/dark cycle with food and water ad libitum. Animal procedures were approved by the TianJin Government Ethics Committees and were in accordance with the Laboratory Animal Science Ethics Requirements of TianJin Medical University. This research was in accordance to the Guide for Laboratory Animal Management and Application printed by China National Health Bureau.

### Complex preparation and identification

The preparation for oxLDL/β2GPI complex was obtained by incubating oxLDL (1 mg apolipoprotein B equivalent/mL) and β2GPI (1 mg/mL) in the absence of CaCl_2_ at 37°C for 16 h. OxLDL/β2GPI (1 mg/mL of apoB equivalent) was further incubated with CRP (0.1 mg/mL) in the presence of 2 mM CaCl_2_ at 37°C for another 16 h to form a nondissociable CRP/oxLDL/β2GPI complex. This procedure was performed as previously described [7].

ELISA for oxLDL/β2GPI complexes. This procedure was performed as previously described [7]. Briefly, anti-β2GPI MAb,WB-CAL-1, was adsorbed onto microtiter plates by incubating at 8 mg/ml (dissolved in Hepes buffer, 50 ml/well) at 4°C over night. After blocking with Hepes buffer containing 1% skim milk, samples diluted 1:100 with Hepes buffer containing 0.5% skim milk were added to the wells (100 ml/well) to be incubated for 2 h. The wells were then incubated with HRP-labeled anti-human apoB-100 MAb (N2E10). Extensive washing between steps was performed with Hepes buffer containing 0.05%Tween20. Color was developed with tetramethylbendizine and H_2_O_2_. The reaction was terminated, and optical density at 450 nm was measured.

ELISA for CRP/oxLDL complex. This procedure was also performed as previously described [7]. Captured anti-apoB-100 MAb,N2E10, was adsorbed onto microtiter plates by incubating at 8 mg/ml (dissolved in Hepes buffer, 50 ml/well) at 4°C overnight. After blocking with 10 mM Tris, 150 mM NaCl, 1.25 mM CaCl2, pH7.4, Tris buffer containing 0.5% BSA, samples diluted 1:100 with Tris buffer containing 0.2% BSA were added to each well and incubated for 2 h. The wells were subsequently incubated with HRP-labeled anti-CRP Abs for 1 h. Extensive washing between steps was performed with Tris buffer containing 0.05% Tween 20. Further steps were performed as described above for oxLDL/β2GPI complex.

### Animal model

Mice were randomised into diabetes mellitus group (DM group, n=120) and normal control group (NC group, n=24) maintained on high-fat and sugar diet composed of (by mg) 10% sugar, 10% lard, 5% yolk, 1% cholesterol, and 0.2% bile salt and standard chow diet, respectively. Each mouse remained on the assigned diet throughout the whole experiment. After eight weeks, the DM group was intraperitoneally injected with 80 mg/kg 2% STZ three times for three days. The tail vein blood glucose was measured 72 h after the injection, and those with blood glucose ≥16.7 mmol/L were considered DM mice. The NC group was simultaneously injected with sodium citrate buffer. Two weeks later, the DM group was treated with oxLDL 20 μg, β2GPI 20 μg, oxLDL/β2GPI complex 40 μg, CRP/oxLDL/β2GPI complex 44 μg, and PBS, after which they were randomly divided into oxLDL group, β2GPI group, oxLDL/β2GPI group, CRP/oxLDL/β2GPI group, and PBS group, respectively (each group n=24). The same interventions were boosted four weeks later. NC group were injected with PBS at the same time. Body weight was assessed every week. After the injection of STZ, blood glucose was monitored weekly. Four weeks later, blood was obtained via retro-orbital plexus and then sacrificed by cervical dislocation. Aortas were carefully dissected from the iliac bifurcation to the aortic arch and external fatty deposits were removed. Complete aortas were then collected. Some were conserved in -80°C for real-time PCR and Western blot, whereas the others were fixed in 4% (w/v) paraformaldehyde for Sudan IV, H&E, and IH staining.

### Plasma lipid level

Blood samples were centrifuged at 3500 rpm for 5 min at room temperature. Plasma concentration of triglycerides (TG) and total cholesterol (TC) were determined by enzymatic colorimetric assays using an Automatic Biochemical Analyzer (Hitachi Co., Japan).

### En face aorta analysis

The aortas were fixed in 4% (w/v) paraformaldehyde overnight and longitudinally cut open under the microscope, after which they were unfolded flat on the slide with a coverslip on top. After rinsing in 70% (v/v) alcohol, the specimens were stained with Sudan IV solution for 15 min, and then decolorised in 80% (v/v) alcohol for 20 min until the normal tissue turned white. Distilled water was used to wash the tissues gently. Filter papers were used to adsorb the liquid. Neutral resins blocked the specimens. Pictures were taken using a PowerShot S70 camera (Canon, Japan) connected to an IX51 microscope (Olympus, Japan). Image analysis was performed with Image Pro-Plus 6.0 (Media Cybernetics, Bethesda, MD). The percentage of plaque coverage was also calculated.

### Histopathology

Paraffin-embedded aortas were serially sectioned at 5 μm thickness, and then deparaffinated with dimethylbenzene twice for 15 min and absolute ethyl alcohol dehydration twice for 5 min, followed by ethanol dehydration, distilled water washing, and haematoxylin staining for 5 min each. Afterwards, water washing, 1% hydrochloric acid ethanol differentiation, water washing, and 0.5% eosin staining for 1 min to 3 min were performed. Next, the aortas were washed with distilled water, 80% ethanol, 95% ethanol, and 100% ethanol, and then transferred with dimethylbenzene twice for 2 min and blocked with neutral resins. Slices were examined by microscopy to evaluate the overall architecture with attention to atherosclerotic changes, in addition to any other histopathology alterations. Measurements of intima thickness were normalised by the media thickness at the thickest area of plaque by Image Pro-Plus 6.0.

### Immunohistochemistry

Paraffin-embedded slices were deparaffinated, dehydrated, washed, and dried successively. After antigen retrieval and blocking, primary antibodies were added (Mac 1:200 or α- SMA 1:100 or CD3 1:100), and then incubated at 4°C overnight. Biotinylated second antibody (1:200) was added and washed with PBS, followed by a 20 min HRP-labelled streptavidin incubation. Infiltrations of macrophages, SMCs, and T cells in aortas were quantified as the percentage of the luminal surface covered by positive cells to plaque area, as previously described [32].

### Quantitative real-time PCR

Primers for scavenger receptor B1 (SR-B1), scavenger receptor B (CD36), ATP binding cassette receptor D1 (ABCD1), ATP binding cassette receptor G1 (ABCG1), and β-actin were designed according to the GenBank database using Primer Express software. The primer sequences for SR-B1 were as follows: forward, 5^′^TTTGGAGTGGTAGTAAAAAGGGC-3^′^ reverse 5^′^-TGACATCAGGGACTCAGAGTAG-3^′^; for CD36, forward 5^′^-GAACCACTGCTTTCAAAAACTGG-3^′^, reverse 5≠-TGCTGTTCTTTGCCACGTCA-3^′^; for ABCA1, forward 5^′^-AGTGATAATCAAAGTCAAAGGCACAC-3^′^, reverse 5^′^-AGCAACTTGGCACTAGTAACTCTG-3^′^; for ABCG1, forward 5^′^-TTCATCGTCCTGGGCATCTT-3^′^, reverse 5^′^-CGGATTTTGTATCTGAGGACGAA-3^′^, for β-actin, 5^′^-TGGAGAAGAGCTATGAGCTGCCTG-3^′^, reverse 5^′^-GTGCCACCAGACAGCACTGTGTTG-3^′^. Total RNA was isolated using Trizol. Reverse transcription for complementary DNA synthesis and analysis were performed as previously described [33]. The reaction system was then prepared for amplification. Folds= 2 ^–ΔΔCt^, ΔΔCt= (Ct1-Ct2)-(Ct3-Ct4). Ct1 and Ct2 are critical cycle numbers of the target gene and the β-actin in the DM group, respectively; Ct3 and Ct4 stand for critical cycle numbers of the target gene and the β-actin in group N, respectively.

### Western blot

The aortas were dissolved and the concentration of protein was determined by the BCA reagents according to the manufacturer's manual. We prepared 10% (w/v) sodium dodecyl sulfate polyacrylamide gel electrophoresis, and then 50 μg protein, 3 μL l mol/L DTT, the same volume of 2× buffer, and enough TBE formed the loading solutions. The initial voltage was 60 V, which changed to 80 V when the sample entered the separation gel. Protein was transferred to the nitrocellulose membrane for 2 h, and the membrane was successively incubated at room temperature with 5% (w/v) bovine serum albumin (BSA) in Tris-buffered saline with Tween 20 (TBST) for 1 h. The membranes were incubated overnight at 4°C with anti-phospho-MKK 3/6, anti-phospho-p38 MAPK antibody, and β-actin at a dilution of 1:1000 in TBST. Membranes were washed with TBST 3 times for 10 min each, and then incubated with a 1:1000 dilution of anti-rabbit horseradish peroxidase antibody for 1 h in the dark. During the end of incubation, the membrane was extensively washed with TBST. The immunoreactive bands were detected by ECL reagents, imaged, and analysed using BandScan software.

### Protein chip

The aortas were dissolved and the protein concentration was determined by BCA reagents as above. Mouse angiogenesis antibody array G Series 1 consists of 24 cytokine antibodies spotted in duplicates on a glass slide [34]. According to the manufacturer’s instructions, the glass slide was assembled into an incubation chamber and dried for 2 h. Samples were added into each well, incubated, and blocked. Biotin-conjugated antibodies and fluorescent dye-conjugated streptavidin were then added. The chamber was covered with adhesion film and then incubated. The slide was placed in a centrifuge full of wash buffer, where it was gently shaken. It was then centrifuged at 4°C and thoroughly dried. A laser scanner (Scan-array Gx, PerkinElmer) was used to read the signals. For each spot, the net density gray level was determined by subtracting the background gray levels from the total raw density gray levels. The relative fold differences in cytokine amount were determined by an analysis tool (Raybio, USA), and graphs were automatically generated.

### Statistical analysis

SPSS18.0 software was used to analyse data. Comparisons among groups were accomplished by one-way ANOVA and Student Newman Keuls (SNK) Test was used for the statistical analysis of any 2 groups. Results were presented as mean ± SD ($\bar{x}$ ± S). Significance was considered when *P* < 0.05, α=0.05.

## Abbreviations

AS: Atherosclerosis; STZ: Streptozotocin; SMCs: Smooth muscle cells; CVD: Cardiovascular diseases; bFGF: Basic fibroblast growth factor; IGF-α: Insulin-like growth factor α

## Competing interests

The authors declare that they have no competing interests.

## Authors’ contributions

Design of the study: RZ, SJZ, DMY, PY; conduct of the study: RZ, SJZ, CJL, XNW; data collection: CJL, XNW, YZT, RC, LL, QZ, QLX; data analysis: CJL, XNW, YZT, RC, LL, QZ, QL X; manuscript writing: RZ, SJZ DMY, PY; final approval: RZ, SJZ, CJL, XNW, YZT, RC, LL, QZ, QLX, DMY, PY.
